# In Vitro Cytotoxic and Inflammatory Response of Gingival Fibroblasts and Oral Mucosal Keratinocytes to 3D Printed Oral Devices

**DOI:** 10.3390/polym16101336

**Published:** 2024-05-09

**Authors:** Maximilian Kollmuss, Daniel Edelhoff, Falk Schwendicke, Sabina Noreen Wuersching

**Affiliations:** 1 Department of Conservative Dentistry and Periodontology, University Hospital, LMU Munich, Goethestrasse 70, 80336 Munich, Germany; falk.schwendicke@med.uni-muenchen.de (F.S.); sabina.wuersching@med.uni-muenchen.de (S.N.W.); 2 Department of Prosthetic Dentistry, University Hospital, LMU Munich, Goethestrasse 70, 80336 Munich, Germany; daniel.edelhoff@med.uni-muenchen.de

**Keywords:** CAD/CAM, 3D printing, apoptosis, oxidative stress, inflammatory response, cell viability

## Abstract

The purpose of this study was to examine the biocompatibility of 3D printed materials used for additive manufacturing of rigid and flexible oral devices. Oral splints were produced and finished from six printable resins (pairs of rigid/flexible materials: KeySplint Hard [KR], KeySplint Soft [KF], V-Print Splint [VR], V-Print Splint Comfort [VF], NextDent Ortho Rigid [NR], NextDent Ortho Flex [NF]), and two types of PMMA blocks for subtractive manufacturing (Tizian Blank PMMA [TR], Tizian Flex Splint Comfort [TF]) as controls. The specimens were eluted in a cell culture medium for 7d. Human gingival fibroblasts (hGF-1) and human oral mucosal keratinocytes (hOK) were exposed to the eluates for 24 h. Cell viability, glutathione levels, apoptosis, necrosis, the cellular inflammatory response (IL-6 and PGE_2_ secretion), and cell morphology were assessed. All eluates led to a slight reduction of hGF-1 viability and intracellular glutathione levels. The strongest cytotoxic response of hGF-1 was observed with KF, NF, and NR eluates (*p* < 0.05 compared to unexposed cells). Viability, caspase-3/7 activity, necrosis levels, and IL-6/PGE_2_ secretion of hOK were barely affected by the materials. All materials showed an overall acceptable biocompatibility. hOK appeared to be more resilient to noxious agents than hGF-1 in vitro. There is insufficient evidence to generalize that flexible materials are more cytotoxic than rigid materials. From a biological point of view, 3D printing seems to be a viable alternative to milling for producing oral devices.

## 1. Introduction

Computer-aided design (CAD) and computer-aided manufacturing (CAM) technologies have revolutionized the way dentistry is practiced today. In modern dental laboratories, most dental restorations are routinely fabricated with subtractive manufacturing techniques using dental milling units. A wide variety of biomaterials can be processed with this method, including glass ceramics, zirconia, metal alloys, and several kinds of polymers. Particularly the use of polymer-based materials has been rapidly evolving over the past few years, mainly driven by the commercialization of additive manufacturing machines, which are commonly referred to as 3D printers. The basic principle of 3D printing is that a liquid photosensitive resin is selectively exposed to the light of a specific wavelength that locally cures the resin, at which point a build platform ascends. This process is repeated several times until the desired object is formed [1]. Nowadays, the most commonly used printing technology is digital light processing (DLP), where an entire layer of resin is exposed to the light, thus leading to optimized manufacturing times [2]. This technique requires novel resin compositions that have not been previously used in dentistry and must be thoroughly examined to judge their clinical applicability. Owing to the ability to create intricate shapes and geometries with high precision, 3D printing is used for rapid prototyping of complex dental restorations, such as crowns, bridges, veneers, and implant abutments, but also for larger devices including dentures, surgical guides, and all kinds of oral appliances [3]. CAD/CAM technologies are progressively replacing traditional manufacturing techniques given their rapid manufacturing time and lower number of time-consuming steps requiring personnel, while providing restorations with high accuracy regarding their internal and occlusal fit [4]. While subtractive CAD/CAM materials are routinely used for fabricating permanent fixed dental prostheses, the long-term use of 3D printing materials for permanent restorations is still limited by their mechanical and esthetic properties [5,6].

Oral appliances are used for a wide range of applications including splints such as occlusal relation splints in patients with temporomandibular disorders, preventive splints in patients with bruxism, or orthodontic retention splints, but also aligners, surgical guides, bleaching or fluoride trays, and athletic mouthguards [7,8,9,10,11,12]. A wide variety of biomaterials intended for additive manufacturing of oral appliances have been introduced and most manufacturers offer a selection of both rigid and flexible materials. The term “flexible” usually refers to materials with thermo-flexible properties or with a thermo-active memory. This feature describes the ability of a material to become pliable when exposed to higher temperatures and to return to its original state upon cooling [13]. The use of flexible materials is justified by the premise that the thermo-active properties facilitate insertion into the oral cavity, leading to enhanced wearing comfort and improved patient compliance [14].

Given the rising public awareness of potential health hazards related to resin-based materials, the use of 3D-printed materials in the oral cavity has raised concerns about the possible toxicity of leaching components. While there are several understandings of biocompatibility, the most suitable definition with respect to oral appliances is the ability of a material to exist in harmony with living cells without causing deleterious changes and while supporting their biological functioning [15,16]. Deleterious changes may include impaired cell viability, induction of oxidative stress and apoptosis, or inflammation [17,18]. Oral appliances present a potential source of leaching monomers which, if present in saliva, may exert such adverse effects on the cells forming the oral mucosa, the two main cell types being keratinocytes and fibroblasts [19]. The surface and wearing time of oral appliances may also affect monomer extraction, considering that oral appliances are rather large and are usually worn for several hours a day.

In recent years, several materials for 3D printing oral devices using DLP printers have been introduced. To date, there is only few data about the biological properties of 3D-printed oral appliances. While there are some studies that examined the toxicity of 3D-printed fixed dental prostheses, there are currently no studies about the toxic and inflammatory potential of 3D-printed oral splints. Therefore, this study aimed to evaluate the cytotoxic and inflammatory effects of recently introduced printable resins for manufacturing oral appliances with DLP printers. In particular, this study analyzed the effects of DLP printing materials on gingival fibroblasts and oral epithelial keratinocytes, as well as the resins’ potential to induce oxidative stress, apoptosis, and necrosis within these cells. The secondary aim of this study was to investigate, whether there is a difference in biocompatibility between rigid and flexible splints.

## 2. Materials and Methods

### 2.1. Materials

Eight materials used for manufacturing flexible and rigid oral appliances were examined in this study: six different printable resins (three flexible, three rigid) intended for additive manufacturing and two types of PMMA blocks (one flexible, one rigid) used for subtractive manufacturing (control groups). Table 1 shows an overview of all materials along with the abbreviations used from this point on. Detailed information about the type and chemical composition of each material is also displayed in Table 1.

### 2.2. Cells

Cytotoxic evaluations of the materials were performed using human gingival fibroblasts (hGF-1, LGC Standards, Wesel, Germany) and immortalized normal human oral mucosal keratinocytes (hOK, Applied Biological Materials Inc., Richmond, BC, Canada). hGF-1 were cultured in high glucose (4.5 g/L) Dulbecco’s Modified Eagle’s Medium (DMEM, gibco/life technologies, Carlsbad, CA, USA) supplemented with 10% fetal bovine serum (FBS, Merck, Darmstadt, Germany) and 100 U/mL penicillin G and 100 µg/mL streptomycin (Merck). hOK were grown in CnT-Prime Epithelial Proliferation Medium (EPM, CELLnTEC, Bern, Switzerland) supplemented with 100 U/mL Penicillin G and 100 µg/mL streptomycin (Merck). Both cell lines were incubated at 37 °C in a 100% humidified atmosphere containing 5% CO_2_ and grown to 70% confluency prior to further subculturing for each experiment.

### 2.3. Resin Sample and Eluate Preparation

A template in the shape of an oral splint was designed using a CAD software (Tizian Creativ RT Version 3.2 Elefsina, Schütz Dental, Rosbach, Germany) and exported as a standard tessellation language (stl) file (Supplementary Figure S1). KR, KF, VR, and VF specimens were manufactured in a DLP printer (P30+, RapidShape, Heimsheim, Germany). NR and NF were produced in a different 3D printer (NextDent 5100, NextDent, Soesterberg, The Netherlands). Both printers were set to a z-axis resolution of 50 µm and the objects were manufactured in a printing orientation of 0° to the build platform. TR and TF specimens were manufactured from a PMMA block with a wet milling protocol in a dental milling unit (CORiTEC 350i, imes-icore, Eiterfeld, Germany). Finishing of the oral splints was performed by a master dental technician on the same day the specimens were produced. All oral splints were post-processed according to the manufacturers’ instructions and finished with the same method following a standard protocol which is routinely used in dental laboratories: a crosscut tungsten carbide bur was first used for surface grinding and smoothing the edges of the splint. Pre-polishing was performed in a dental polishing unit with a pumice powder and liquid mixture (Steriwet, Benzer Dental, Zurich, Switzerland). Detailed specifications for the manufacturing and post-processing of each material are shown in Table 1. On the following day, the oral splints were disinfected in 80% ethanol for 1 min and left to air-dry. Potential bacteria colonizing the surface of the oral splints were assumed to be eliminated after this disinfection protocol [20]. Eluate preparation was performed for each material and cell line by submerging one entire oral splint in 50 mL of the appropriate cell culture medium (DMEM and EPM) and incubating them for 7 days at 37 °C and 100% humidity. The eluates were sterilized by passing them through a 0.2 µm pore membrane filter (VWR International, Darmstadt, Germany) and then stored at 4 °C until further use. This elution procedure was performed with four separate sets of oral splints to obtain four individual replicates for each cell line.

### 2.4. Exposure of Cells to Eluates

Approximately 6 × 10^4^ hGF-1 and hOK were seeded into the wells of a 6-well plate and incubated overnight for adherent growth. After removing the cell culture medium from the wells, the adherent cells were treated with the prepared eluates as well as with three further solutions serving as positive control groups for the subsequent assays: a 1 µM Staurosporin solution (STS, Merck, control group for apoptosis and necrosis), a 1 µM Bortezumib solution (BZM, Merck, control group for cell proliferation), and a 1 µM Lipopolysaccharide solution (LPS from *Escherichia coli* O127:B8, Merck, control group for cellular inflammatory response). Cell culture medium served as the negative control group. The cells were incubated with the eluates and the solutions for the control groups for 24 h. The supernatants were collected for later detection of inflammatory mediators. The treated cells were trypsinized (Trypsin/EDTA solution 2.5%, Merck) and resuspended in a cell culture medium. The resuspended cells were used for determining cell viability, necrosis, and GSH levels. For determining the early release of caspase enzymes during apoptosis, the cells were only incubated for 5 h.

### 2.5. Cell Viability

An ATP-based cell viability assay was performed to measure the number of metabolically active hGF-1 and hOK (CellTiterGlo 2.0 Assay, Promega, Madison, WI, USA) after exposure to the eluates. The assay reagent was prepared according to the manufacturer’s recommendations and added to the cell suspension to lyse the cells and release their intracellular ATP. Upon exposure to ATP, the assay returned a luminescence signal (relative light units, RLU) which was measured in a luminometer (GloMax Navigator System, Promega). Cell viability was computed as the percentage of the control group (cell culture medium containing no agent).

### 2.6. Necrosis

After an incubation time of 24 h with the eluates, necrotic cells were detected using a fluorescent dye which stains cellular DNA, indicating cells with loss of membrane integrity (Celltox Green Cytotoxicity Assay, Promega). Fluorescence was recorded in terms of relative fluorescence units (RFU) at 485–500 nm_Ex_/520–530 nm_Em_ (Varioskan, Thermo Fisher Scientific, Waltham, MA, USA).

### 2.7. Apoptosis

The occurrence of apoptosis was detected after 5 h incubation of the cells with the eluates by measuring the caspase-3 and caspase-7 activity within the cells using a luminescence-based apoptosis assay (Caspase Glo 3/7 Assay, Promega). Cell culture medium served as the negative control and the apoptosis inducer staurosporine (STS, Merck) as the positive control. The assay reagent containing a luminogenic caspase-3/7 substrate was prepared according to the protocol and added to the cells. The activated caspases-3 and -7 in cells undergoing apoptosis cleave the substrate, generating a luminescence signal (RLU) which was measured in a luminometer (GloMax Navigator System).

### 2.8. Glutathione Levels

A luminescence-based assay was performed to measure the concentrations of reduced glutathione (GSH) and oxidized glutathione (GSSG) within the cells (GSH/GSSG-Glo™ Assay, Promega). All reagents were prepared according to the protocol and added to the cells. After 15 min incubation at room temperature, a luminescence signal (RLU) proportional to the GSH and GSSG concentration was measured in a luminometer. For converting the RLUs to GSH and GSSG concentrations, a standard curve describing the relationship between luminescence and the glutathione concentration was generated.

### 2.9. Cell Morphology

The cell morphology of adherent hGF-1 and hOK exposed to the eluates and to the control groups for 24 h was examined with a phase contrast microscope. Microscopic images of the cells were taken at a 100× magnification (Axiovert 40 C, Axiocam 305 color, Carl Zeiss, Oberkochen, Germany).

### 2.10. Inflammatory Response

The presence of the inflammatory mediators interleukin 6 (IL-6) and prostaglandine E_2_ (PGE_2_) within the supernatants was used as measure of cellular inflammation after exposure to the eluates. IL-6 and PGE_2_ concentrations were determined with a luminescence-based immunoassay (Lumit IL-6 Immunoassay, Promega) and a specific enzyme-linked immunosorbent assay (PGE_2_ high sensitivity ELISA kit, Enzo life Sciences, Lörrach, Germany) according to the manufacturer’s instructions. Standard curves were generated to calculate the IL-6 and PGE_2_ concentrations. To exclude interferences of the ELISA detection kit with the eluates, additional standards were performed with each oral splint eluate serving as a diluent. 

### 2.11. Statistics

All experimental conditions were performed four times in independent experiments. Statistical analyses were implemented in Python 3.11. The packages *scipy* and *scikit* were used for inferential statistics and *matplotlib* was used for the descriptive analyses [21]. Homoscedasticity was assessed with Levene’s test and data were tested for normality with the Shapiro–Wilk test. For normally distributed data with equal variances, comparisons between groups were performed using a one-way analysis of variances (ANOVA) and Tukey’s post hoc analysis. Normally distributed data with unequal variances were analyzed with Welch’s ANOVA and Tamhane’s T2 post hoc analysis. The alpha level was set to 0.05.

## 3. Results

### 3.1. Cell Viability

**Figure 1:**
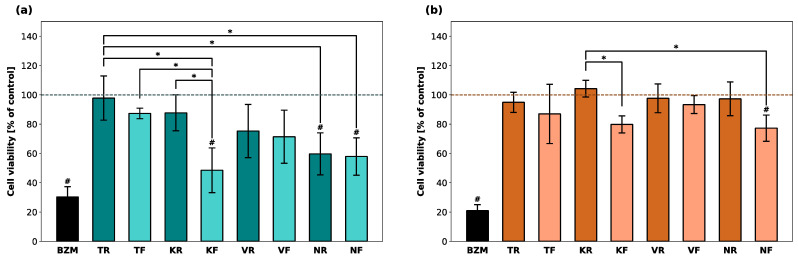
Cell viability of hGF-1 (**a**) and hOK (**b**) after exposure to eluates of 3D-printed oral appliances. Data are shown as a percentage of the no-treatment control group (dashed line). *p*-values obtained with ANOVA and Tukey’s post hoc test. ^#^, *p* < 0.05 compared to the no-treatment control group with cell culture medium (DMEM/EPM); *, *p* < 0.05 between groups indicated with brackets. Significant differences between BZM and splint groups are not displayed.

The cell viability of hGF-1 and hOK after incubation with the eluates of the splint materials and with BZM (positive control) was computed as the percentage of the control group (untreated cells) and is displayed in Figure 1. BZM reduced the proportion of viable hGF-1 and hOK cells to 30% and 20%, respectively. Among the hGF-1 cells incubated with the eluates, TR, TF, and KR eluates led to the highest average percentage of viable cells, whereas the lowest percentage was detected after incubation with KF, NR, and NF eluates. The KF, NR, and NF groups showed significantly lower cell viability than at least one other material group. As far as the cell viability of hOK cells is concerned, KF and NF were the only materials that led to a significantly lower cell viability compared to another group. Cell viability in the presence of all other materials was in a similar range as untreated hOK cells.

### 3.2. Necrosis

**Figure 2:**
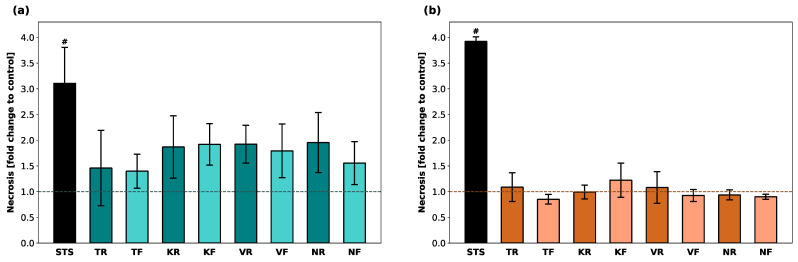
Occurrence of necrosis in hGF-1 (**a**) and hOK (**b**) after exposure to eluates of 3D-printed oral appliances. Data shown as fold change of relative fluorescence units (RFU) compared to the control group (dashed line). *p*-values obtained with ANOVA and Tukey’s post hoc test. ^#^, *p* < 0.05 compared to the no-treatment control group with cell culture medium (DMEM/EPM). Significant differences between STS and splint groups are not displayed.

Fluorescence levels (RFUs) as an indicator of necrosis in hGF-1 and hOK are shown in terms of fold change compared to untreated cells (Figure 2). Exposure of hGF-1 to STS (positive control) led to a 3-fold increase in necrosis levels. In general, all splint materials led to enhanced necrosis levels in hGF-1, however, there was no statistically significant increase compared to the control group. For cells incubated with KR, KF, VR, VF, and NR eluates, an average 2-fold increase in fluorescence was detected. Among the experimental groups, the lowest necrosis activity of hGF-1 was recorded after exposure to the TR, TF, and NF eluates. STS caused a 4-fold increase in hOK, whereas none of the tested materials led to an increase in RFUs compared to untreated hOK. There were no significant differences in necrosis levels between any of the splint materials.

### 3.3. Apoptosis

**Figure 3:**
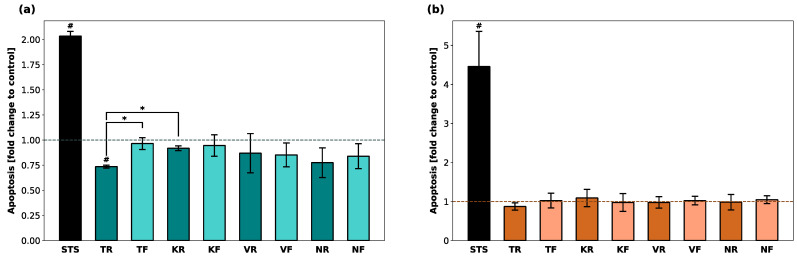
Induction of apoptosis in hGF-1 (**a**) and hOK (**b**) by eluates of 3D-printed oral appliances. Data are shown as a fold change of relative light units (RLU) compared to the control group (dashed line). *p*-values obtained with Welch’s ANOVA and Tamhane’s post hoc test. ^#^, *p* < 0.05 compared to the no-treatment control group with cell culture medium (DMEM/EPM); *, *p* < 0.05 between groups indicated with brackets. Significant differences between STS and splint groups are not displayed.

Luminescence (RLU) indicating the presence of apoptotic cells was calculated as a fold change compared to untreated cells (Figure 3). While STS (positive control) led to a fold change of 2 and 4.5 in hGF-1 and hOK, respectively, no apoptotic activity was detected in cells incubated with any of the material eluates. For both cell lines, the RLUs in all test groups were significantly lower compared to the positive control group. In the presence of TR, apoptosis activity was significantly lower compared to TF and KR.

### 3.4. Glutathione Levels

**Figure 4:**
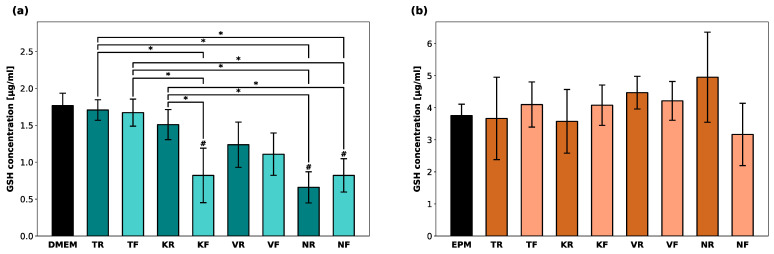
Intracellular glutathione (GSH) concentration [µg/mL] in hGF-1 (**a**) and hOK (**b**) after exposure to eluates of 3D-printed oral appliances. *p*-values obtained with ANOVA and Tukey’s post hoc test. ^#^, *p* < 0.05 compared to the no-treatment control group with cell culture medium (DMEM/EPM); *, *p* < 0.05 between groups indicated with brackets.

No increased GSSG levels were detected for either cell line. Therefore, only the reduced GSH levels in hGF-1 and hOK after exposure to eluates of the oral splints are shown in Figure 4. KR, NR, and NF led to a significant GSH depletion in hGF-1 compared to the negative control group with cell culture medium. GSH levels in hOK were barely affected in the presence of any of the tested materials.

### 3.5. Cell Morphology

**Figure 5:**
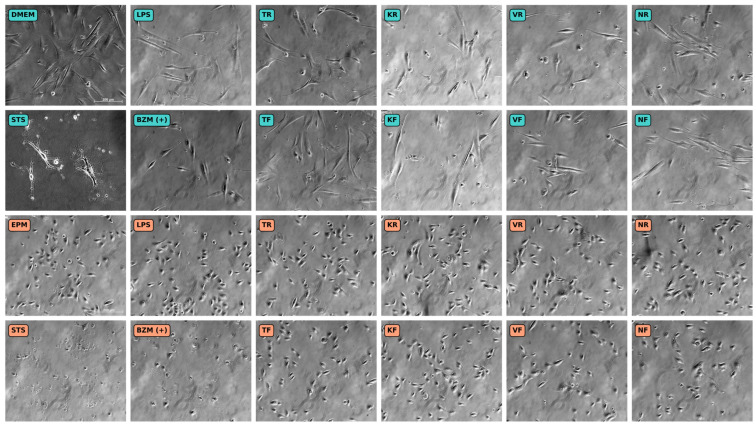
Microscopic images showing the cell morphology of hGF-1 (turquoise) and hOK (orange) after exposure to eluates of 3D-printed oral appliances as well as to no-treatment control with cell culture medium (DMEM/EPM) and positive controls with BZM and STS. hGF-1 and hOK at 15–20 days of in vitro cultivation. Images taken with a phase contract microscope at 100× magnification.

Microscopic images of hGF-1 and hOK after incubation with the material eluates and the solutions for the control groups are shown in Figure 5. hGF-1 cells incubated with DMEM (negative control) showed typical signs of a healthy fibroblast morphology, such as an elongated, stellate cell shape, and their cytoplasm tapered off into long slender processes. The untreated hOK (negative control) exhibited a polygonal cell shape with large nuclei and were arranged in a monolayer. Since hOK were not grown to confluency during the experiment, the cell monolayer did not have the cobblestone-like appearance that is typical for confluent epithelial cells. The presence of BZM (positive control for cell viability) led to a lower cell density in both hGF-1 and hOK compared to the negative control group with the respective cell culture media (DMEM/EPM). LPS (positive control for inflammation) had no impact on the cell morphology and the density of both the hGF-1 and hOK. STS (positive control for apoptosis and necrosis) caused both cell types to detach from the culture surface and led to typical signs of cell death, such as loss of cell membrane integrity, cytoplasmic shedding, cell shrinkage, and cell fragmentation with the formation of discrete bodies. In the presence of the eluates obtained from the 3D-printed materials (KR, KF, VR, VF, NR, and NF), hGF-1 exhibited shorter processes and a rounded cell shape, indicating possible growth arrest and inactive cell metabolism. Cell density in the groups with the 3D-printed splint eluates seemed lower compared to the negative control group with DMEM. As far as hOK are concerned, the overall morphology and cell density was barely affected by any of the tested splint materials. However, for both cell types the microscopic images reveal occasional cell swelling or shrinkage amid the intact cells.

### 3.6. Inflammatory Response

**Figure 6:**
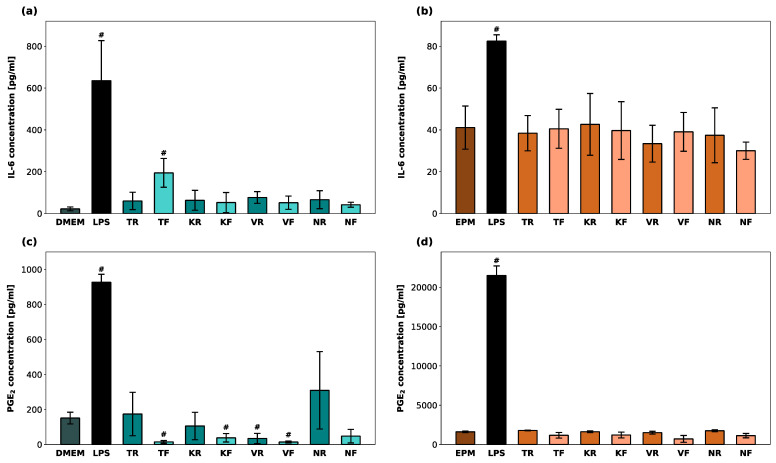
Inflammatory response in hGF-1 (**a**,**c**) and hOK (**b**,**d**) after exposure to eluates of 3D-printed oral appliances. Data represent IL-6 and PGE_2_ concentrations [pg/mL] within the supernatants. *p*-values obtained with ANOVA and Tukey’s post hoc test (**a**,**b**) and with Welch’s ANOVA and Tamhane’s post hoc test (**c**,**d**). ^#^, *p* < 0.05 compared to the no-treatment control group with cell culture medium (DMEM/EPM). Significant differences between LPS and splint groups are not displayed.

Cellular IL-6 and PGE_2_-levels within hGF-1 and hOK after exposure to the material eluates as well as to 1 µM of LPS (positive control) are shown in Figure 6. LPS led to a significant increase in IL-6 and PGE_2_ production in both cell types (*p* < 0.05). IL-6 levels in hGF-1 were significantly increased only in the presence of TF. TF, KF, VR, and VF led to significantly lower PGE_2_ levels in hGF-1 compared to the untreated cells (*p* < 0.05). No significant changes in IL-6 and PGE_2_-levels in hOK were observed among the test groups and the negative control group.

## 4. Discussion

Additive CAD/CAM technologies are on the rise in dentistry and are progressively replacing traditional manufacturing techniques. Particularly for occlusal splint fabrication, the photopolymerization techniques using DLP and stereolithography (SLA) printers have become popular due to several advantages related to customization, reproducibility, and throughput [1,2]. The emerging use of 3D-printed oral devices in dentistry requires thorough examinations of the biological interactions between the materials and host cells. According to the ISO recommendations for in vitro cytotoxicity testing of medical devices, the extracting conditions for obtaining liquid extracts from a material should simulate or exaggerate the clinical conditions of use to demonstrate the potential danger of the material being tested [22]. Therefore, we used an entire average-sized occlusal splint instead of small specimens such as discs or cubes which are frequently used in similar studies. This approach was based on the premise that the release of leaching components is dependent on the size and the surface properties of the material. For example, the use of standardized discs does not consider the fact that for clinical use the intaglio surface of oral splints is usually not polished, which gives the device a different surface structure compared to a perfectly polished surface [23].

For the purpose of investigating possible cytotoxic and pro-inflammatory effects of the oral splint materials in this study, fibroblasts and keratinocytes were chosen, since they are the two main cell types forming the oral mucosa. Being part of the stratified squamous epithelium, which is the outermost layer of the oral mucosa and gingiva, hOK are the first cells exposed to any kinds of stimuli in the oral cavity. However, noxious agents may cause adverse effects on the underlying tissue either by diffusing through the epithelial barrier or by directly interacting with the tissue when exposed due to injuries or during surgery. Oral fibroblasts are the most abundant cell types present in the connective and supporting tissues of the oral cavity. Fibroblasts exhibit a broad set of biological functions, which include defining the tissue architecture, producing extracellular matrix, eliciting host defense mechanisms, and supporting wound healing processes and remodeling of injured tissues [24,25,26]. Based on their clinical significance to oral splints, both hGF-1 and hOK cells were deemed suitable for testing the cytotoxic and pro-inflammatory effects of the materials in this study.

Noxious agents can cause oxidative stress, a cellular response which is characterized by the intracellular accumulation of reactive oxygen species (ROS) [27]. To protect the DNA from oxidative damage, cells are supplied with ROS scavengers such as GSH that act as a biological redox buffer. Upon exposure to oxidizing agents, GSH is converted into its oxidized form GSSG. Thus, the cellular balance of GSH and GSSG serves as a dynamic indicator of oxidative stress, and the GSH/GSSG ratio can be used for judging the redox status within the cells. As long as an equilibrium between oxidation and reduction is maintained, GSSG levels increase, while the respective GSH levels simultaneously decrease [28]. When the detoxifying ability of GSH is exhausted, the total GSH concentration decreases, which activates apoptosis pathways and ultimately leads to secondary necrosis [29]. However, in the case of hGF-1 exposed to KF, NR, and NF eluates, GSH levels were significantly lower compared to untreated hGF-1, but there was no simultaneous increase in GSSG. Results from a similar in vitro biocompatibility study suggest that 3D-printed oral splint materials cause intracellular ROS accumulation in cells [30]. Furthermore, previous studies have demonstrated that TEGDMA and HEMA, two monomers commonly used in resins for 3D printing oral splints, can cause intracellular GSH depletion and cell damage to gingival fibroblasts [31,32]. In particular, HEMA-induced oxidative stress has been shown to occur through covalent binding to GSH and subsequent GSH-HEMA complex formation, which eventually leads to an exhaustion of the intracellular GSH pool [33]. Similar mechanisms have also been reported for other methacrylates commonly found in 3D-printed materials.

Since the total glutathione concentration, which encompasses both redox forms of glutathione, is proportional to the cell number, our results suggest that there were fewer hGF-1 after exposure to KF, NR, and NF. This supposition is also in agreement with the significantly lower ATP levels registered in the presence of these three materials. However, further biological responses must be taken into consideration to identify the reasons for the lower number of viable cells, as the ATP and GSH/GSSG data alone do not suffice for this purpose. The occurrence of apoptosis was assessed by determining the presence of caspase-3 and caspase-7 with a luminogenic assay. The activation of both caspases, which are the executioners of the caspase-dependent apoptosis pathway, commits a cell to programmed death [34]. Being late effectors within the apoptosis cascade, caspase 3- and 7 can be activated by both the intrinsic and extrinsic apoptosis pathway. We opted for an assay targeting a late event of the cascade to confirm that the cells actually underwent apoptosis regardless of the preceding mechanism. While the apoptosis inducer STS led to a significant increase in caspase-3 and -7 activity in hGF-1, there were no signs of apoptosis after incubation with the oral splint eluates. The absence of GSSG within all oral splint groups corresponds with the absence of apoptotic cells. For detecting necrotic cells, a fluorogenic assay marking cells with loss of membrane integrity was performed. Although the increase in RFU was not significant compared to the control group, our results suggest that the presence of the 3D-printed oral splint eluates caused a 1.5 to 2-fold increase in necrotic hGF-1 compared to unstimulated cells. Furthermore, morphological analyses show that the overall cell density was lower after incubation with the oral splint eluates compared to untreated hGF-1. Altogether, it is fairly unlikely that the hGF-1 were affected by ROS accumulation, apoptosis, or secondary necrosis. Based on our results, we may assume that the main reason for the lower cell number registered for some of the groups was inhibited cell proliferation, accompanied by the fact that a certain proportion of cells underwent primary necrosis. It appears as though the TR and TF eluates, which served as the control group for materials processed via subtractive manufacturing, had the least effect on hGF-1 in terms of viability and GSH levels. TR and TF also caused the least morphological changes to hGF-1 compared to untreated cells. These findings are also in accordance with our previous work, which implied a lower cytotoxicity of dental crowns milled from PMMA blocks compared to crowns made via additive manufacturing [35]. The lower toxicity of milled specimens was attributed to the industrial polymerization procedure of the PMMA blocks, which is performed under high temperature and high pressure to optimize the degree of monomer conversion. Indeed, it has been previously shown that a higher degree of conversion is associated with lower cytotoxicity due to fewer residual monomers [36,37]. As far as the hOK is concerned, all data related to viability, GSH and necrosis indicate an overall low cytotoxic response to all oral splint materials. Although the microscopic images reveal occasional signs of apoptosis, such as cell swelling and shrinkage, no increased caspase-3 and -7 activity was registered for the hOK. As opposed to hGF-1, there was no association between the cytotoxic response of hOK and the type of manufacturing procedure. In general, the hOK appeared to be more resilient to toxic stimuli than the hGF-1. This finding is plausible given that the biological purpose of keratinocytes is to create a sufficient barrier against possible noxious agents to protect the underlying connecting tissue. Conversely, when the epithelial barrier is damaged, for example due to injuries or after surgical procedures, the presence of toxic stimuli may affect the dynamics of wound healing and tissue remodeling which are regulated by the hGF-1 [25].

In addition to identifying cytotoxic effects, a further essential part of biocompatibility testing is assessing the presence of inflammation within the host cells. IL-6 is a pro-inflammatory cytokine that marks the early stages of inflammation and is promptly produced by various cell types including fibroblasts and epithelial cells in response to toxic stimuli and tissue injuries [38,39]. In addition to functioning as a mediator during the acute phase of inflammation, IL-6 is involved in regulating the transition between acute and chronic inflammation [40]. Dysregulated and persistent synthesis of IL-6 can initiate chronic inflammatory responses, thus favoring the development or disease progression of gingivitis and periodontitis in the oral cavity [41]. PGE_2_ is a derivative of arachidonic acid and is synthesized by the cyclooxygenases COX-1 and COX-2. Some of the characteristic biological functions of PGE_2_ are pyrexia, pain sensation, and inflammation. With regard to the oral cavity, the cyclooxygenase pathways of inflammation generating PGE_2_ due to a COX-2 overexpression have been shown to encourage the development and progression of oral cancer, including oral squamous cell carcinoma [42]. To our knowledge, the cellular inflammatory response to 3D-printed oral splints has not been previously examined. However, there are a few studies that have investigated cellular inflammation in the presence of 3D-printed dental restorations [35,43]. A frequent finding in these studies was that cells exposed to these materials produced fewer cytokines than untreated cells, but the overall inflammatory effects were moderate. Our data implies that hOK did not show any in vitro inflammatory response to the oral splint eluates in terms of IL-6 secretion or PGE_2_ upregulation. The relatively high basal level in untreated hGF-1 and hOK is probably attributed to the constitutive expression of COX-1 [44]. As far as the hGF-1 is concerned, only TF eluates led to an increase in IL-6 concentration within the supernatants. One main difference between TF and TR regarding their composition is the incorporation of a plasticizer into the PMMA matrix of TF. 1,2-Cyclohexane dicarboxylic acid diisononyl ester (DINCH), which is responsible for the flexible properties in polymerized TF, emerged as an alternative to former plasticizers that have been restricted due to recurrent health concerns. However, recent studies have reported that DINCH exerts pro-inflammatory effects on macrophages [45]. Perhaps the enhanced IL-6 secretion by the hGF-1 after incubation with the TF eluates was also attributed to the pro-inflammatory nature of DINCH. To confirm the presence of DINCH in the eluates, detailed analyses of the extraction liquids are required. Interestingly, the PGE_2_ concentration was reduced in the presence of TF, KF, VR, and VF. A possible explanation for this finding is that certain components within the eluates acted as COX inhibitors. Nonetheless, it should be mentioned that the concentrations of the inflammatory mediators detected within the supernatants are dependent on the total cell number. Since the cell number of hGF-1 appears to have been affected by the presence of some eluates, our data may underestimate the IL-6 and PGE_2_ concentrations for these groups, assuming that there is a linear proportion between the cell number and cytokine release. However, both IL-6 and PGE_2_ are relatively stable in vitro and may have been produced by cells that turned necrotic at some point [46,47]. Thus, the true concentration of the secreted inflammatory mediators cannot be determined by simply normalizing the data to the number of cells, which limits the significance of our results.

Having insight into the chemical composition of the materials is important for judging the clinical applicability of medical devices from a biological point of view. In general, resins used for additive manufacturing of oral devices comprise different types of photopolymerizing methacrylates and a photoinitiator. As opposed to the printable materials used for manufacturing dental crowns and bridges, the oral splint materials do not contain any inorganic filler particles such as silanized dental glass [35]. According to the available information provided by the manufacturers, the printable oral splint materials used in this study contain the Norrish type I photoinitiator diphenyl (2,4,6-trimethylbenzoyl) phosphine oxide (TPO). Phosphine oxide photoinitiators are commonly used for 3D printing due to their excellent degree of conversion, high polymerization rate, and good color stability [48]. Nonetheless, on account of previous in vitro data indicating that TPO exerts genotoxic and cytotoxic effects on keratinocytes and fibroblasts, there have been recurrent concerns about unreacted TPO leaching out into the oral cavity [49,50,51]. It is important to note that these studies used pure solutions of TPO to treat the cells, which is a valid procedure for demonstrating the potential toxicological hazard of TPO; however, these results may also overestimate the actual toxicity of certain components in clinical conditions of use. In fact, recent investigations have shown that polymerized resins containing TPO as a photoinitiator show excellent biocompatibility [52]. In a previous study that examined liquid extracts of 3D-printed oral splints via gas chromatography/mass spectrometry (GC/MS) analysis, TPO was not detected as a leaching compound despite being a component of the material [19]. The composition of the eluates was not analyzed in this study, and it is therefore uncertain whether TPO was present within the in vitro eluates or was responsible for any damage to the cells. However, since all eluates showed an overall low cytotoxicity, despite the ratio between specimen size and extraction liquid reflecting exaggerated conditions, one may assume that TPO did not leach out in significant concentrations given that the material is sufficiently polymerized. Naturally, this is a mere speculation that requires further analytical studies for confirmation. To demonstrate the hazardous effects of TPO with reference to clinical conditions, one would have to first determine the release kinetics of TPO from each material, for example, using GC/MS, and then use TPO concentrations that are assumed to be present in saliva for treating the cells. However, this approach would require a different elution procedure from the one used in this study.

As a secondary aim of this study, we investigated whether there are any differences in biocompatibility between rigid and thermo-flexible splints. To our knowledge, this study objective has not been addressed before. In our previous work, we demonstrated that oral splints with thermoplastic properties exhibit a higher surface roughness, making them more susceptible to bacterial adhesion and biofilm formation [23]. Based on our results from this present study, there are only minor implications referring to the idea that flexible oral splints may be less biocompatible than rigid ones. Our main findings supporting this thesis are the significant differences registered between KF and KR regarding cell viability and intracellular GSH levels, as well as the fact that cytokine secretion from hGF-1 was higher with TF than with TR. Regarding the apoptosis and necrosis activity, no difference between rigid and thermo-flexible materials was registered. Altogether, our results do not provide sufficient evidence to draw a general conclusion that flexible oral splint materials are more cytotoxic than rigid materials.

As for every study, there are some limitations that should be mentioned. Although this research was designed to emulate typical clinical conditions in the oral cavity, our data are limited by the in vitro nature of this study, which is unable to replicate the complex environment in the human body. Several mechanisms related to toxicity and inflammation cannot be simulated in an in vitro study. Moreover, the choice of liquid used for elution was limited by the requirement for the extraction medium to be compatible with the cells used for the in vitro testing. However, the composition of the cell culture media is quite different from saliva in terms of protein content, which may affect the extraction and binding of leaching components. A further limitation of this study is that the oral splints were not subjected to artificial aging or cyclic fatigue. Since oral devices are periodically exposed to mastication in the oral cavity, the type and quantity of components leaching out into saliva may be different in vivo. It should be mentioned that all data implying toxicity reflect a worst-case scenario considering that leaching components are immediately diluted by saliva in the oral cavity. Altogether, our in vitro results provide the foundation for future studies wanting to evaluate the long-term biocompatibility of the oral splint materials.

## 5. Conclusions

This study provides a comprehensive comparison of the biological properties of 3D-printed oral devices manufactured using DLP printers. Within the general limitations of an in vitro study, all oral splint materials showed overall acceptable biocompatibility. Our results indicate that the oral splints fabricated with subtractive manufacturing techniques elicited the weakest cytotoxic response of hGF-1. There is no sufficient evidence to support the thesis that rigid and flexible oral splint materials show differences in biocompatibility. A key finding of this study is that oral keratinocytes are more resilient to noxious agents than fibroblasts in vitro. From a methodological point of view, this highlights the importance of choosing suitable cell types for in vitro studies with regard to their clinical significance. Further studies are needed to provide more detailed insight into the release of leaching components from these materials and the effects of each released component on the host cells.

## Figures and Tables

**Table 1:** Oral splint materials were used in this study. Manufacturing technique and post-processing steps according to the manufacturer’s instructions. Abbreviations with R = rigid material, F = flexible (thermoplastic) material. Chemical composition of each material according to the information provided by the manufacturers.

Material	Abbr.	Manufacturer	Manufacturing Details	Composition	LOT-No.
Tizian Blank PMMA	TR	Schütz Dental, Rosbach, Germany	milled from a 98 mm diameter milling blank in imes.icore 450i, polishing with pumice/water	PMMA	41522
Tizian Flex Splint Comfort	TF	Schütz Dental	milled from a 98 mm diameter milling blank in imes.icore 450i, polishing with pumice/water	>90% PMMA, <10% DINCH	160520211
KeySplint Hard	KR	Keystone Industries, Gibbstown, NJ, USA	3D printing in rapidshape P30, wash/cure with rapidshape system (setting for KeySplint Hard), polishing with pumice/water	Mixture of methacrylates *	LF0778
KeySplint Soft	KF	Keystone Industries	3D printing in rapidshape P30, wash/cure with rapidshape system (setting for KeySplint Soft), polishing with pumice/water	Mixture of methacrylates *	MH5425
V-Print Splint	VR	Voco, Cuxhaven, Germany	3D printing in rapidshape P30, wash/cure with rapidshape system (setting for V-Print Splint), polishing pumice/water	Polyesterdimethacrylate, Bis-EMA, TEGDMA, HPMA, BHT, TPO	2236383
V-Print Splint Comfort	VF	Voco	3D printing in rapidshape P30, wash/cure with rapidshape system (setting for V-Print Comfort), polishing pumice/water	Aliphatic acrylate, TEGDMA, TPO	2247279
NextDent Ortho Rigid	NR	NextDent, Soesterberg, The Netherlands	3D-printing in NextDent 5100, wash 2 times (3 min + 2 min) in alcohol, cure 30 min at 60 °C (LC-3D Print Box) polishing pumice/water	Bis-EMA, methacrylic oligomer, HEMA; 7,7,9(or 7,9,9)-trimethyl-4,13-dioxo-3,14-dioxa-5,12-diazahexadecane-1,16-diyl bismethacrylate, TPO	WW232N04
NextDent Ortho Flex	NF	NextDent	3D printing in NextDent 5100, wash 2 times (3 min + 2 min) in alcohol, cure 10 min at 60 °C (LC-3D Print Box) polishing pumice/water	2-phenoxyethyl acrylate; 4-(1-oxo-2-propenyl)-morpholine; methacrylate ester monomer; TPO; acrylate ester	WW285N02

* further specifications remained undisclosed. Abbreviations: PMMA—polymethylmethacylate; DINCH—1,2-Cyclohexane dicarboxylic acid diisononyl ester; Bis-EMA—bisphenol A ethoxylate dimethacrylate; TEGDMA—triethylene glycol dimethacrylate; HPMA—hydroxypropyl methacrylate; BHT—butylated hydroxytoluene; TPO—diphenyl (2,4,6-trimethylbenzoyl) phosphine oxide, HEMA—hydroxyethyl methacrylate.

## Data Availability

The raw data supporting the conclusions of this article will be made available by the authors on request.

## Supplementary Materials

The following supporting information can be downloaded at: https://www.mdpi.com/article/10.3390/polym16101336/s1, Figure S1: STL data of the oral splint used for additive and subtractive manufacturing of the study specimens.

## References

[1] Liaw C.-Y., Guvendiren M. (2017). Current and Emerging Applications of 3D Printing in Medicine. Biofabrication.

[2] Dawood A., Marti B.M., Sauret-Jackson V., Darwood A. (2015). 3D Printing in Dentistry. Br. Dent. J..

[3] Tian Y., Chen C., Xu X., Wang J., Hou X., Li K., Lu X., Shi H., Lee E.-S., Jiang H.B. (2021). A Review of 3D Printing in Dentistry: Technologies, Affecting Factors, and Applications. Scanning.

[4] Reymus M., Hickel R., Kessler A. (2020). Accuracy of CAD/CAM-Fabricated Bite Splints: Milling vs 3D Printing. Clin. Oral Investig..

[5] Vichi A., Balestra D., Scotti N., Louca C., Paolone G. (2023). Translucency of CAD/CAM and 3D Printable Composite Materials for Permanent Dental Restorations. Polymers.

[6] Jain S., Sayed M.E., Shetty M., Alqahtani S.M., Al Wadei M.H.D., Gupta S.G., Othman A.A.A., Alshehri A.H., Alqarni H., Mobarki A.H. (2022). Physical and Mechanical Properties of 3D-Printed Provisional Crowns and Fixed Dental Prosthesis Resins Compared to CAD/CAM Milled and Conventional Provisional Resins: A Systematic Review and Meta-Analysis. Polymers.

[7] Christidis N., Lindström Ndanshau E., Sandberg A., Tsilingaridis G. (2019). Prevalence and Treatment Strategies Regarding Temporomandibular Disorders in Children and Adolescents-A Systematic Review. J. Oral Rehabil..

[8] Beddis H., Pemberton M., Davies S. (2018). Sleep Bruxism: An Overview for Clinicians. Br. Dent. J..

[9] Rossini G., Parrini S., Castroflorio T., Deregibus A., Debernardi C.L. (2015). Efficacy of Clear Aligners in Controlling Orthodontic Tooth Movement: A Systematic Review. Angle Orthod..

[10] Johnston C.D., Littlewood S.J. (2015). Retention in Orthodontics. Br. Dent. J..

[11] Unkovskiy A., Huettig F., Kraemer-Fernandez P., Spintzyk S. (2021). Multi-Material 3D Printing of a Customized Sports Mouth Guard: Proof-of-Concept Clinical Case. Int. J. Environ. Res. Public Health.

[12] Sliwkanich L., Ouanounou A. (2021). Mouthguards in Dentistry: Current Recommendations for Dentists. Dent. Traumatol..

[13] Perea-Lowery L., Gibreel M., Garoushi S., Vallittu P., Lassila L. (2023). Evaluation of Flexible Three-Dimensionally Printed Occlusal Splint Materials: An in Vitro Study. Dent. Mater..

[14] Herpel C., Kykal J., Rues S., Schwindling F.S., Rammelsberg P., Eberhard L. (2023). Thermo-Flexible Resin for the 3D Printing of Occlusal Splints: A Randomized Pilot Trial. J. Dent..

[15] Vert M., Doi Y., Hellwich K.-H., Hess M., Hodge P., Kubisa P., Rinaudo M., Schué F. (2012). Terminology for Biorelated Polymers and Applications (IUPAC Recommendations 2012). Pure Appl. Chem..

[16] Schmalz G., Arenholt-Bindslev D. (2008). Biocompatibility of Dental Materials.

[17] Wuersching S.N., Högg C., Kohl L., Reichl F.-X., Hickel R., Kollmuss M. (2023). Leaching Components and Initial Biocompatibility of Novel Bioactive Restorative Materials. Dent. Mater..

[18] Yang Y., Reichl F.X., Shi J., He X., Hickel R., Högg C. (2018). Cytotoxicity and DNA Double-Strand Breaks in Human Gingival Fibroblasts Exposed to Eluates of Dental Composites. Dent. Mater..

[19] Wedekind L., Güth J.F., Schweiger J., Kollmuss M., Reichl F.X., Edelhoff D., Högg C. (2021). Elution Behavior of a 3D-Printed, Milled and Conventional Resin-Based Occlusal Splint Material. Dent. Mater..

[20] Morton H. (1950). The Relationship of Concentration and Germicidal Efficiency of Ethyl Alcohol. Ann. N. Y. Acad. Sci..

[21] van Rossum G., Drake F. (2019). Python 3 Reference Manual.

[22] (2009). Biological Evaluation of Medical Devices—Part 5: Tests for in Vitro Cytotoxicity.

[23] Wuersching S.N., Westphal D., Stawarczyk B., Edelhoff D., Kollmuss M. (2023). Surface Properties and Initial Bacterial Biofilm Growth on 3D-Printed Oral Appliances: A Comparative in Vitro Study. Clin. Oral Investig..

[24] Darby I.A., Hewitson T.D. (2007). Fibroblast Differentiation in Wound Healing and Fibrosis. Int. Rev. Cytol..

[25] Bainbridge P. (2013). Wound Healing and the Role of Fibroblasts. J. Wound Care.

[26] Tzach-Nahman R., Nashef R., Fleissig O., Palmon A., Shapira L., Wilensky A., Nussbaum G. (2017). Oral Fibroblasts Modulate the Macrophage Response to Bacterial Challenge. Sci. Rep..

[27] Krifka S., Seidenader C., Hiller K.A., Schmalz G., Schweikl H. (2012). Oxidative Stress and Cytotoxicity Generated by Dental Composites in Human Pulp Cells. Clin. Oral Investig..

[28] Jones D.P. (2002). [11] Redox Potential of GSH/GSSG Couple: Assay and Biological Significance. Methods in Enzymology.

[29] Turrens J.F. (2003). Mitochondrial Formation of Reactive Oxygen Species. J. Physiol..

[30] Guerrero-Gironés J., López-García S., Pecci-Lloret M.R., Pecci-Lloret M.P., Rodríguez Lozano F.J., García-Bernal D. (2022). In Vitro Biocompatibility Testing of 3D Printing and Conventional Resins for Occlusal Devices. J. Dent..

[31] Volk J., Engelmann J., Leyhausen G., Geurtsen W. (2006). Effects of Three Resin Monomers on the Cellular Glutathione Concentration of Cultured Human Gingival Fibroblasts. Dent. Mater..

[32] Stanislawski L., Lefeuvre M., Bourd K., Soheili-Majd E., Goldberg M., Périanin A. (2003). TEGDMA-Induced Toxicity in Human Fibroblasts Is Associated with Early and Drastic Glutathione Depletion with Subsequent Production of Oxygen Reactive Species. J. Biomed. Mater. Res. Part A.

[33] Gallorini M., Cataldi A., di Giacomo V. (2014). HEMA-Induced Cytotoxicity: Oxidative Stress, Genotoxicity and Apoptosis. Int. Endod. J..

[34] Elmore S. (2007). Apoptosis: A Review of Programmed Cell Death. Toxicol. Pathol..

[35] Wuersching S.N., Hickel R., Edelhoff D., Kollmuss M. (2022). Initial Biocompatibility of Novel Resins for 3D Printed Fixed Dental Prostheses. Dent. Mater..

[36] Barreto Girão L., Ohana de Lima Martins J., Lemos J.V.M., Pinto M.R., Rolim J.P.M.L., Alves e Silva F.C.F., Saboia V.D.P.A., Bitu Sousa F., de Barros Silva P.G. (2020). Influence of the Degree of Conversion and Bis-GMA Residues of Bulk Fill Resins on Tissue Toxicity in an Subcutaneous Model in Rats. J. Appl. Biomater. Funct. Mater..

[37] Fujioka-Kobayashi M., Miron R.J., Lussi A., Gruber R., Ilie N., Price R.B., Schmalz G. (2019). Effect of the Degree of Conversion of Resin-Based Composites on Cytotoxicity, Cell Attachment, and Gene Expression. Dent. Mater..

[38] Tanaka T., Narazaki M., Kishimoto T. (2014). IL-6 in Inflammation, Immunity, and Disease. Cold Spring Harb. Perspect. Biol..

[39] Krueger J., Ray A., Tamm I., Sehgal P.B. (1991). Expression and Function of Interleukin-6 in Epithelial Cells. J. Cell. Biochem..

[40] Kaplanski G., Marin V., Montero-Julian F., Mantovani A., Farnarier C. (2003). IL-6: A Regulator of the Transition from Neutrophil to Monocyte Recruitment during Inflammation. Trends Immunol..

[41] Naruishi K., Nagata T. (2018). Biological Effects of Interleukin-6 on Gingival Fibroblasts: Cytokine Regulation in Periodontitis. J. Cell. Physiol..

[42] Abrahao A.C., Castilho R.M., Squarize C.H., Molinolo A.A., dos Santos-Pinto D.J., Gutkind J.S. (2010). A Role for COX2-Derived PGE2 and PGE2-Receptor Subtypes in Head and Neck Squamous Carcinoma Cell Proliferation. Oral Oncol..

[43] Folwaczny M., Ahantab R., Kessler A., Ern C., Frasheri I. (2023). Cytotoxicity of 3D Printed Resin Materials for Temporary Restorations on Human Periodontal Ligament (PDL-HTERT) Cells. Dent. Mater..

[44] DuBois R.N., Abramson S.B., Crofford L., Gupta R.A., Simon L.S., Putte L.B.A., Lipsky P.E. (1998). Cyclooxygenase in Biology and Disease. FASEB J..

[45] Schaffert A., Arnold J., Karkossa I., Blüher M., von Bergen M., Schubert K. (2021). The Emerging Plasticizer Alternative DINCH and Its Metabolite MINCH Induce Oxidative Stress and Enhance Inflammatory Responses in Human THP-1 Macrophages. Cells.

[46] De Jager W., Bourcier K., Rijkers G.T., Prakken B.J., Seyfert-Margolis V. (2009). Prerequisites for Cytokine Measurements in Clinical Trials with Multiplex Immunoassays. BMC Immunol..

[47] Kalinski P. (2012). Regulation of Immune Responses by Prostaglandin E2. J. Immunol..

[48] Meereis C.T.W., Leal F.B., Lima G.S., De Carvalho R.V., Piva E., Ogliari F.A. (2014). BAPO as an Alternative Photoinitiator for the Radical Polymerization of Dental Resins. Dent. Mater..

[49] Wessels M., Rimkus J., Leyhausen G., Volk J., Geurtsen W. (2015). Genotoxic Effects of Camphorquinone and DMT on Human Oral and Intestinal Cells. Dent. Mater..

[50] Van Landuyt K.L., Krifka S., Hiller K.A., Bolay C., Waha C., Van Meerbeek B., Schmalz G., Schweikl H. (2015). Evaluation of Cell Responses toward Adhesives with Different Photoinitiating Systems. Dent. Mater..

[51] Popal M., Volk J., Leyhausen G., Geurtsen W. (2018). Cytotoxic and Genotoxic Potential of the Type I Photoinitiators BAPO and TPO on Human Oral Keratinocytes and V79 Fibroblasts. Dent. Mater..

[52] Kim G.-T., Go H.-B., Yu J.-H., Yang S.-Y., Kim K.-M., Choi S.-H., Kwon J.-S. (2022). Cytotoxicity, Colour Stability and Dimensional Accuracy of 3D Printing Resin with Three Different Photoinitiators. Polymers.

