# Supplementary material for: In Vitro Cytotoxic and Inflammatory Response of Gingival Fibroblasts and Oral Mucosal Keratinocytes to 3D Printed Oral Devices
Source: Polymers (Basel). 2024 May 9;16(10):1336. doi: 10.3390/polym16101336 (PMC11125196; doi:10.3390/polym16101336)
Supplement: Supplementary file 1 [file polymers-16-01336-s001.zip › polymers-2969360-supplementary.pdf]

SUPPLEMENTARY INFORMATION

Article: **In vitro cytotoxic and inflammatory response of gingival fibroblasts and oral mucosal keratinocytes to 3D printed oral devices**

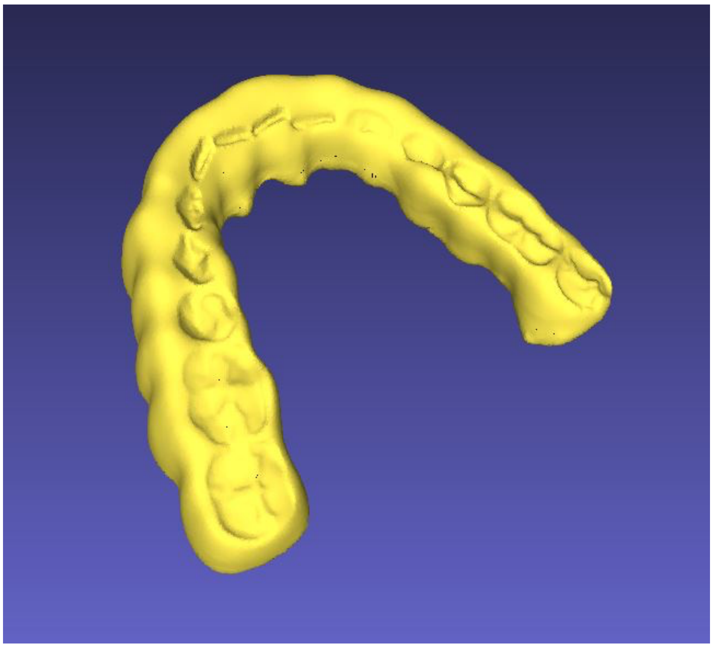

Supplementary Figure S1. STL data of the oral splint used for additive and subtractive manufacturing of the study specimens.
